# Cell cycle-dependent cues regulate temporal patterning of the *Drosophila* central brain neural stem cells

**DOI:** 10.1101/2025.01.16.629716

**Published:** 2025-01-16

**Authors:** Gonzalo N. Morales Chaya, Mubarak Hussain Syed

**Affiliations:** 1 Neural Diversity Lab, Department of Biology, University of New Mexico, Albuquerque, NM 87131, USA; 2 Institute of Neuroscience, Howard Hughes Medical Institute, University of Oregon, Eugene,OR 97403, USA

## Abstract

During nervous system development, diverse types of neurons and glia are sequentially generated by self-renewing neural stem cells (NSCs). Temporal changes in gene expression within NSCs are thought to regulate neural diversity; however, the mechanisms regulating the timing of these temporal gene transitions remain poorly understood. *Drosophila* type II NSCs, like human outer radial glia, divide to self-renew and generate intermediate neural progenitors, amplifying and diversifying the population of neurons innervating the central complex, a brain region required for sensorimotor coordination. Type II NSCs express over a dozen genes temporally, broadly classified as early and late-expressed genes. A conserved gene, Seven-up mediates early to late gene expression by activating ecdysone receptor (EcR) expression. However, what determines the timing of EcR expression and, hence, early to late gene transition is unknown. This study investigates whether intrinsic mechanisms of cell cycle progression and cytokinesis are required to induce the NSC early-late transition. By generating mutant clones that arrest the NSC cell cycle or block cytokinesis, we show that both processes are necessary for the early-to-late transitions. When NSCs are cell cycle or cytokinesis arrested, the early gene Imp failed to be down-regulated and persisted into the old NSCs, while the late factors EcR and Syncrip failed to be expressed. Furthermore, we show that the early factor Seven-up is insufficient to drive the transition despite its normal expression in the cell cycle- or cytokinesis-inhibited NSCs. These results suggest that both intrinsic (cell cycle/cytokinesis) and extrinsic (hormone) cues are required for the early-late NSC gene expression transition.

## Introduction

From insects to humans, multipotent neural stem cells (NSCs) generate diverse neurons and glia, which mediate the proper nervous system function. Both spatial and temporal patterning regulate the diversity of neurons generated during nervous system development (Doe, 2017; El-Danaf et al., 2023; Enriquez et al., 2015; Erclik et al., 2017; Hamid et al., 2023; Kohwi and Doe, 2013; Malin and Desplan, 2021; Oberst et al., 2019; Sen, 2023; Skeath and Thor, 2003). While much is known about the regulation of spatial identity, how temporal changes occur over time is not entirely understood. Understanding the processes through which NSCs undergo temporal patterning, enabling them to produce diverse neurons and glial cells in the nervous system, is critical for understanding brain development and disease. Studies on the Drosophila nervous system have identified temporal identity genes that express temporally in NSCs and diversify neural identity (Bayraktar and Doe, 2013; Eldred et al., 2018; Isshiki et al., 2001; Li et al., 2013; Maurange et al., 2008; Syed et al., 2017; Yang et al., 2015). Similar temporal identity programs were later shown to play a role in the vertebrate retina and cortex cell type specification (Alsiö et al., 2013; Elliott et al., 2008; Javed et al., 2023; Kohwi and Doe, 2013; Koo et al., 2023; Pebworth et al., 2021; Telley et al., 2019). These gene transitions were thought to be intrinsically regulated in Drosophila embryonic NSCs; however, our recent findings in the larval central brain NSCs identified the role of extrinsic steroid growth hormone ecdysone on the early to late gene transition (Syed et al., 2017). Recently, thyroid hormone signaling was identified to regulate the early to late-born cone cell type in human retinal organoid cultures (Eldred et al., 2018). Over the past few years, various temporally expressed genes were identified in both invertebrates and vertebrates; however, what determines the precise timing of these gene transitions is poorly understood.

Based on division patterns, the Drosophila NSCs can be classified as type 0, type I, or type II NSCs. Type 0 NSCs generate a single post-mitotic neuron with each division; type 0 NSCs have only been observed in embryonic stages (Arefin et al., 2020; Baumgardt et al., 2014). Type I NSCs generate a ganglion mother cell (GMC) with each division; each GMC makes two post-mitotic neurons (Doe, 2017; Doe and Goodman, 1985; Rossi and Desplan, 2017). Type I NSCs comprise the majority of NSCs in the brain. Type II NSCs have an even more complex lineage: they generate Intermediate Neural Progenitors (INPs) with each division; INPs undergo a series of 4–6 self-renewing divisions to produce 4–6 GMCs, which each generate two neurons (Bello et al., 2008; Boone and Doe, 2008; Bowman et al., 2008; Wang et al., 2014). Recent temporal RNA-sequencing experiments have revealed that type II NSCs undergo a gene expression cascade of transcription factors (TFs) and RNA-binding proteins (RBPs). In early larvae 0–60h after larval hatching (ALH), the NSCs express TFs, Seven-up (Svp), Castor (Cas), Chinmo, and RBPs, IGF-II mRNA-binding protein (Imp), and Lin-28. In late larvae (60–120h ALH), the NSCs express TFs, Ecdysone receptor (EcR), Ecdysone-induced protein 93F (E93), Broad and RBP, Syncrip (Syp) (Ren et al., 2017; Syed et al., 2017). The early expressed conserved TF, Svp, regulates the expression of the EcR through a yet unknown mechanism.

The activation of EcR renders NSCs competent to respond to the cell-extrinsic ecdysone hormone, which triggers the early-late gene expression switch (Syed et al., 2017). While the role of the extrinsic factor Ecdysone in triggering the early-late transition is understood, nothing is known about how intrinsic factors drive this transition and what regulates the precise timing of these events. In vertebrate cortical development, the cell cycle plays a key role in regulating a neural progenitor’s ability to respond to extrinsic cues. Transplanted progenitors in a late-stage host exhibit different responses to these cues depending on their cell cycle stage at the time of transplantation (McConnell and Kaznowski, 1991; Ohnuma and Harris, 2003). There is precedent for proposing an intrinsic cell cycle-regulated mechanism of larval temporal gene expression: embryonic NSCs use two cell-intrinsic mechanisms for the timely transition of Hunchback > Kruppel > Pdm1/2 > Cas. First, blocking cell cycle progression prevents the Hunchback > Kruppel transition as well as all later genes in the cascade (Grosskortenhaus et al., 2005). Second, blocking cytokinesis (but not nuclear divisions) also stalls the temporal cascade at Hunchback (Grosskortenhaus et al., 2005). Similarly, it has been shown that the NSC transition from early larval Imp/Chinmo to Syp/Broad relies on the energy state of the cell and progression from the G1/S phase (van den Ameele and Brand, 2019). However, whether the switching factors, Svp and EcR, and hence the timing of the transition, are cell-cycle dependent and whether similar mechanisms operate in the central brain type II NSCs is unknown. Moreover, it is unclear whether the timing of NSC gene expression is driven by organismal cues (e.g., hormones or signaling pathways), internal cues (e.g., a cell cycle counting clock), or both. Here, we use larval central brain NSCs to identify the mechanisms that regulate the timing of the temporal gene expression.

## Results

### Cell cycle and cytokinesis are both required for the early-late temporal factor switch in type II NSCs

**Figure 1:**
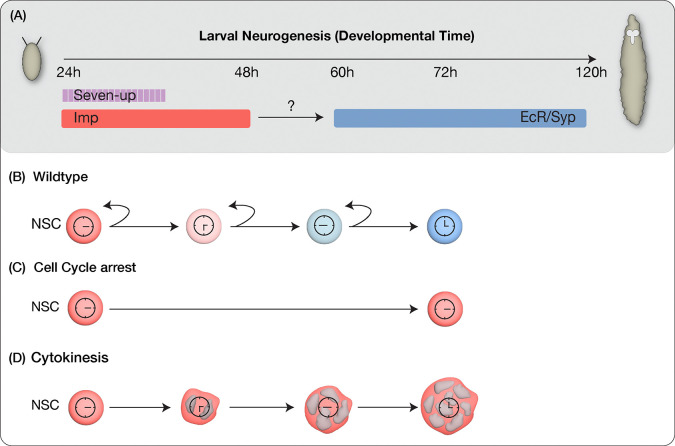
Models for early-to-late temporal progression transition of type II NSCs. (A) Depicts timing of NSC temporal factor expression in type II NSCs during Drosophila larval development (B) Wild type NSCs undergo normal early to late temporal factor progression (C) Cell cycle progression as a regulator to allow timely expression of factors in NSCs (D) Cytokinesis dependent regulation of temporal gene expression. Cell analog clocks indicate the progression (or stop) of cell cycle.

**Figure 2:**
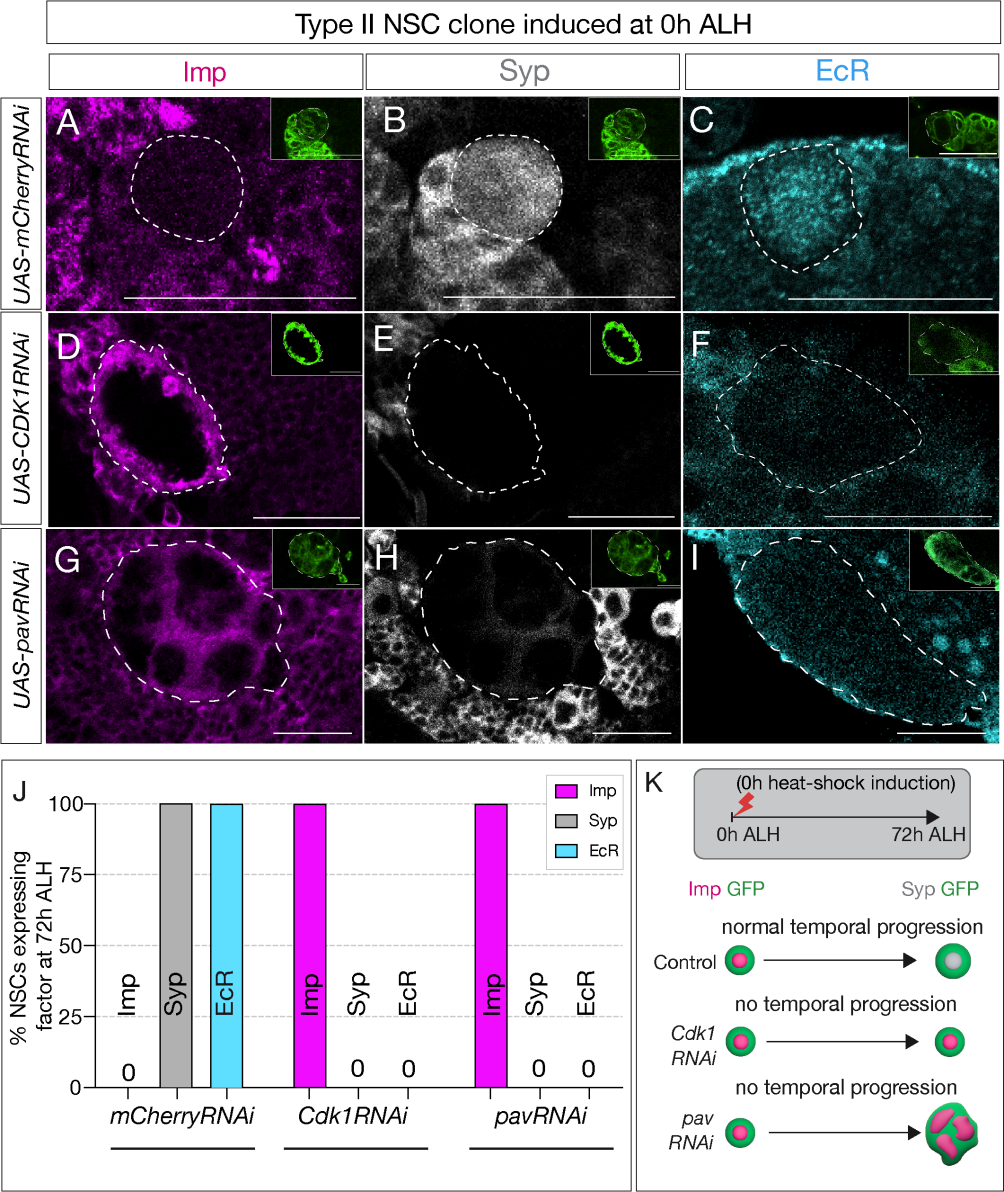
Cell cycle and cytokinesis are required for temporal gene expression progression in type II NSCs. (A-C) Type II NSC control *mCherry*RNAi clones (circled) at 72h ALH show normal temporal gene expression progression. Early factor Imp is off, and late factors Syp and EcR express at 72h ALH (D-F) *Cdk1*RNAi type II NSC clone fail to down-regulate early factor Imp and do not express late factors, Syp and EcR. (G-I) Similarly, cytokinesis-blocked type II NSC clones using *pav*RNAi fail to downregulate Imp and up-regulate Syp and EcR. (J) Quantifications of early and late temporal factor expression in control, cell cycle, and cytokinesis blocked type II NSCs; n>4 for each genotype. (K) Representation of experimental layout for inducing clones at 0h ALH and analysis at 72h ALH. Type II NSCs are identified as Asense negative large cells expressing UAS-mcd8::GFP (green insets). Scale bars represent 20um.

Our previous work revealed that in type II NSCs, the transition from early to late factors occurs around 55h ALH (Syed et al., 2017). Next, we wanted to elucidate the mechanisms governing the precise timing of these gene transition shifts. Previous evidence for intrinsic mechanisms regulating temporal patterning in embryonic type I NSCs led us to ask whether intrinsic cues were also used to drive temporal gene expression in larval type II NSCs, which are known to use extrinsic signals to progress through their TTF cascade (Grosskortenhaus et al., 2005; Syed et al., 2017). It is plausible that various cell-intrinsic factors, including genetic factors and cell cycle timer, might orchestrate the timing of these transitions. To investigate whether the temporal progression of type II NSCs requires cell cycle progression, we blocked the NSC cell cycle transition or cytokinesis (the experimental design is shown in (Figure 1). Cyclin-dependent kinase 1 (Cdk1) is a protein kinase essential for initiating mitosis (M phase) and progressing through the G2 phase in eukaryotic cells. We reduced *Cdk1* expression in type II NSCs and assayed the temporally expressed early and late genes. We achieved this by inducing random clones generated by heat shock at zero hours after larval hatching (0h ALH) using the genotype *hsflp, 10xUAS-mCD8GFP; ActFRT-stopFRTGAL4*. Both control RNAi (*UAS-mCherryRNAi*) and experimental (*UAS-Cdk1RNAi*) clones were labeled by GFP, and type II hits were confirmed by lack of the type I Asense marker. After heat shock, we assayed the clones in 72h ALH larvae. Since NSCs decrease in size as they divide throughout development (Hartenstein et al., 1987), the efficacy of the *Cdk1*RNAi in blocking cell cycle was confirmed by measuring cell size, which showed a significant increase in mutant versus control clones (Figure S1). In the control type II NSCs, as anticipated at 72h ALH, minimal or no expression of the early factor Imp was observed, alongside high expression of late factors EcR and Syp (Figure 2A–2C; quantified in Figure 2J). In contrast, the experimental type II NSCs, where the cell cycle was blocked, exhibited persistent expression of the early factor Imp and no expression of late factors EcR and Syp at 72h ALH (Figure 2D–2F; quantified in Figure 2J), thus affirming that cell cycle progression is indispensable for the early-late switch in temporal factor gene expression.

The observed lack of early to late gene transition could stem from either a lack of nuclear divisions (potentially part of a gene expression “clock” or “timer”) or due to a lack of feedback signaling from the NSC progeny, which is not generated following cell cycle block. To discern between these possibilities, we inhibited NSC cytokinesis, halting NSC progeny production while leaving nuclear divisions unaffected (Grosskortenhaus et al., 2005). To do this, we induced clones using *pavarotti* RNAi (*UAS-pavRNAi*), a known inhibitor of cytokinesis that, when knocked down, produces large cells with multiple nuclei (Adams et al., 1998). Control type II NSCs showed no or little Imp expression and downregulation and EcR/Syp (Figure 2A–2C; quantified in 2J), while the experimental *pav*RNAi type II NSCs resulted in large multi nucleated NSCs (Figure S1 C–C’) that maintained Imp expression and lacked EcR/Syp expression (Figure 2 G–2I; quantified in 2J). We conclude that both nuclear division and cytokinesis are essential for the early-late gene expression switch in type II NSCs (Figure 2K), and they may be timed by feedback from the NSC newborn and adjacent progeny.

### The switching factor Svp is normally expressed in cell-cycle arrested NSCs

**Figure 3:**
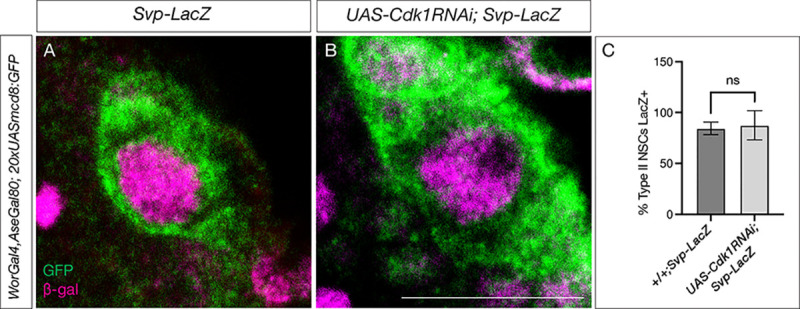
Switching factor Svp expresses normally in cell-cycle arrested type II NSCs. (A) Control type II NSCs marked in green show Svp-LacZ expression at 48h ALH. (B) Cell-cycle arrested (*Cdk1*RNAi) type II NSCs expression Svp similar to the control (C) Quantification of LacZ expression in control and cell cycle blocked type II NSCs, n<10 for each genotype. Scale bars represent 10um.

A conserved nuclear hormone receptor, Svp, mediates the early to late gene switch by regulating EcR expression (Dillon et al., 2024, Maurange et al., 2008, Syed et al., 2024). The *svp* mutant type II NSCs stay in their early gene expression module and fail to exit the cell cycle at the end of larval life. The mutant type II NSCs fail to down-regulate the expression of early factors Chinmo and Imp and show no expression of the late factors EcR, Broad, Syncrip, and E93. While Svp shows its peak expression at 36h ALH, EcR expression starts at around 55h ALH (Dillon et al., 2024, Ren et al. 2017, Syed et al., 2017). Although *svp* is required for EcR expression, what regulates the precise timing of EcR expression around 55h ALH is unknown. To test whether cell-cycle-arrested NSCs fail to express Svp and thus produce a phenotype similar to the *svp* mutant, we assayed Svp expression in type II cell-cycle-arrested NSCs. Since Svp expression is transient in these NSCs, we used a *svpLacZ* line to achieve more stable protein expression. By 48h ALH, nearly all control type II NSCs showed *svpLacZ* expression (Figure 3A), and cell-cycle-arrested animals displayed similar expression levels, with no statistically significant difference from controls (Figure 3B, quantified in 3C). We conclude that the timing of Svp expression is normal in cell-cycle-arrested NSCs.

### Svp and cell cycle progression are independently required for the early-late temporal factor switch

We next wondered whether the cell cycle acts as a timer for the precise EcR expression following Svp expression. To address this question, we generated type II NSC clones expressing either *Cdk*1RNAi or *pav*RNAi at 42h ALH (see methods), after the Svp expression window (36h ALH), and assayed larvae for expression of NSC temporal markers at 72h ALH. If the transition fails to occur, it will confirm that Svp primes EcR expression, but the intrinsic cell cycle clock determines the precise timing. Conversely, if the transition occurs normally, cell cycle/cytokinesis plays a role in early gene expression (before 48h), but not for later post-Svp expression.

**Figure 4:**
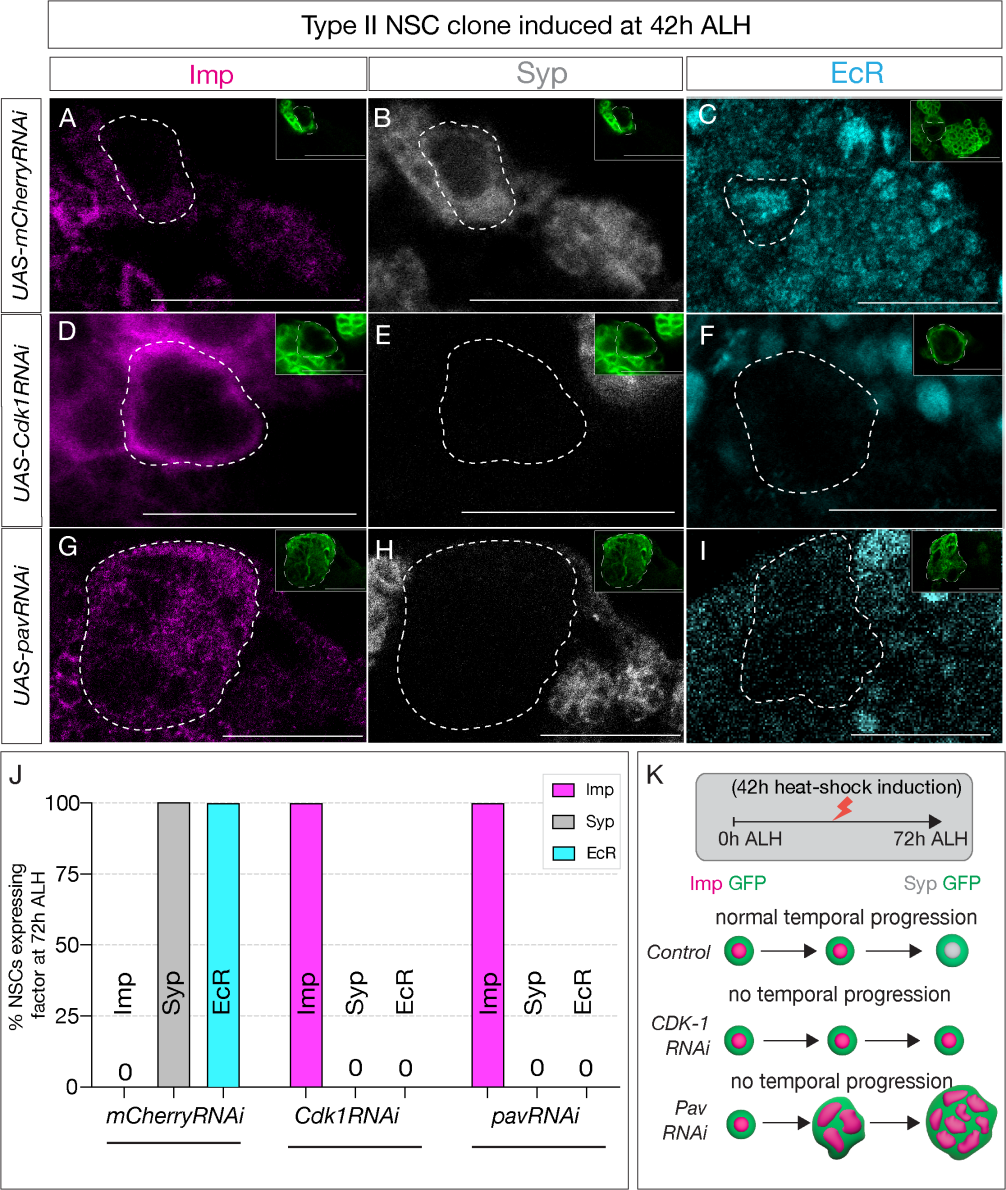
Early Svp expression is not sufficient to drive temporal factor progression in type II NSCs. (A-C) Type II NSC control *mCherry*RNAi clones (circled) induced at 42h ALH and stained at 72h ALH show normal progression of temporal factors. Early factor Imp is off and late factors Syp and EcR are on. (D-F) *Cdk1*RNAi clones show consistent failure to down-regulate early factor Imp and activate Syp and EcR (G-I) Likewise, *pav*RNAi clones fail to express EcR and Syp and consistently express ear;y factor, Imp. (J) Quantification of early and late expressed factors in control, Cdk1 and *pav*RNAi type II NSCs, n>4 for each genotype. (K) Representation of experimental layout. Clones are identified as Asense negative large cells expressing UAS-mcd8::GFP (green insets). Scale bars represent 20um.

Compared to control type II NSC clones that showed normal Imp downregulation and EcR/Syp expression at 72h ALH (Figure 4A–4C; quantified in 4J), *Cdk1* and *pav* mutant type II NSC clones had persistent Imp expression and no EcR/Syp expression (Figure 4D,4I; quantified in 4J), confirming that cell cycle progression is essential for precise timing of EcR and early to late gene transition (Figure 3K).

### Cell cycle and cytokinesis are required for the early-late temporal factor switch in type I NSCs

So far, we have focused on type II NSCs, which are only 16 in number and generate INPs to produce complex lineages. However, most larval NSCs are type I NSCs, expressing the same early and late temporal factors as type II NSCs. The timing of EcR expression and early to late gene transition also matches the type II NSC pattern (Syed et al., 2017). Thus, we wanted to know if cell cycle and cytokinesis also regulate temporal gene transition in Type I NSCs.

**Figure 5:**
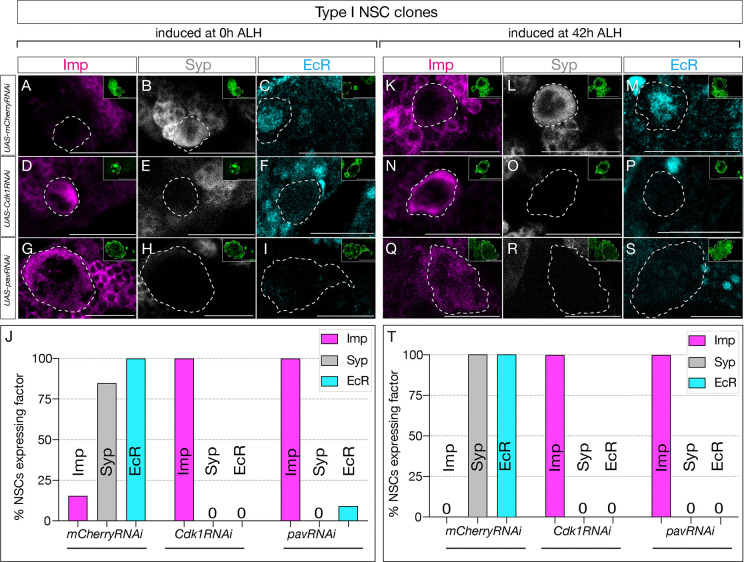
Cell cycle and cytokinesis are required for temporal progression in type I NSCs. (A-I). Clones were induced at 0h ALH, and for (K-S), clones were induced at 42h ALH. (A-C) Type I NSC control RNAi clones show normal expression of temporal factor progression. (D-F) Cell cycle blocked type I NSC clones using *Cdk1*RNAi show failure to up-regulate EcR and Syp and consistent expression of early factor Imp. (G-I) *pav*RNAi type I NSC clones fail to downregulate early factor Imp and late factors Syp and EcR fail to express. (K-M) Control type 1 NSC clones induced at 42h ALH show normal expression of early and late factors, while *Cdk1*RNAi clones (N-P) and *pav*RNAi clones (Q-S) fail to downregulate Imp and fail to activate late factors Syp and EcR. (J, T) Quantification for early and late factor expressions of 0h and 42h ALH-induced clones. For each genotype n>8. Type I clones are identified as Asense positive large cells expressing mcd8::GFP (green insets). Scale bars represent 20um.

Similar to the type II NSC experiments, we generated clones using the genotype *hsFLP, UAS-mC-D8GFP ; ActFRTstopFRTGAL4 / UAS-Cdk1RNAi or UAS-mCherryRNAi* and assayed GFP and Asense positive type I NSCs. Control RNAi induced at 0h and assayed at 72h showed normal down-regulation of Imp and expression of the late factors Syp (Figure 5A–C and 5K–M; quantified in 5J and 5T). In contrast, *Cdk1* or *pav*RNAi type I NSc clones induced at 0h and assayed at 72h maintained Imp expression and lacked EcR/Syp expression (Figure 5D–I; quantified in 5J).

Similarly, *Cdk1* or *pav*RNAi induced at 42h -- after the Svp expression window -- and assayed at 72h also maintained Imp expression and lacked EcR/Syp expression (Figure 5N–S; quantified in 5T). This suggests that most central brain neural stem cells possess a cell-cycle and cytokinesis-dependent, cell-intrinsic mechanism that regulates the Imp/Syp expression transition. Our studies indicate that all central brain NSCs require cell cycle and cytokinesis together with the switching factor Svp expression to drive the early-late temporal factor expression transition.

## Discussion

Temporal patterning plays a crucial role in specifying the diverse cell types of the nervous system. While many temporally expressed genes have been identified in both Drosophila and vertebrate neural progenitors (Javed et al., 2023; Koo et al., 2023; Pebworth et al., 2021; Syed et al., 2017; Telley et al., 2019; Walsh and Doe, 2017), what governs the timing of these gene expression transitions is not clearly understood. Is there an intrinsic timer within NSCs that counts and regulates the precise timing of temporal gene transitions? Is such a mechanism linked to cell-cycle progression and cytokinesis? How are NSC intrinsic and extrinsic environmental cues coupled? This study investigated the cell-intrinsic mechanisms regulating the early-to-late temporal gene expression transitions. Using Drosophila larval central brain NSCs, we identified that both cell cycle and cytokinesis-dependent cell-intrinsic timer regulate the progression of gene expression. In cell cycle, cytokinesis blocked NSCs, transition from early Imp to late EcR and Syp expression was impeded in both type I and type II NSCs. Upon temporal cell cycle block at 42h ALH, we showed that normal cell cycle progression is still essential for EcR expression despite the Svp expression being normal in the NSCs. These findings provide insights into the possible mechanisms regulating the temporal gene transition timing and also reveal the complex interplay of the cell cycle, cell-intrinsic genes, and cell-extrinsic hormonal cues in determining the precise timing of neuron type production within neural progenitors.

The possible role of cell cycle and cytokinesis in regulating temporal patterning and neuronal identity has been studied in vertebrates and invertebrates (Doe, 2008; Grosskortenhaus et al., 2005). In vertebrates, the G2/M phase is required for the sequential generation of layer-specific cortical neurons. Knockdown of G2/M regulators has been shown to prevent radial glial cells from maintaining a “young,” multipotent state, leading to premature differentiation and a bias toward the production of early-born neurons (Hagey et al., 2020; Kawaguchi, 2019). In the case of Drosophila embryonic NSCs, which sequentially express Hunchback, Kruppel, Pdm1, and Castor, the Hunchback to Kruppel expression requires cytokinesis, while the Kruppel to Pdm1 to Castor transition occurs normally in G2 arrested NSCs (Grosskortenhaus et al., 2005). In contrast, our studies on the larval NSCs show that cell cycle and cytokinesis are both essential for the early Imp/Chinmo to late EcR/Syncrip gene transition. The embryonic NSC temporal gene progressions occur normally in isolated NSCs, suggesting intrinsic only regulation of these gene transitions. In contrast, larval NSCs express EcR and require extrinsic growth hormone, ecdysone, for the early to late gene transitions. Our findings suggest that NSCs with more complex division patterns and dividing in situations where the growth and division patterns need to be coordinated require both cell-intrinsic and extrinsic cues.

Through the cell cycle, neural progenitors generate neurons populating distinct layers in the vertebrate cortex (Borrell and Calegari, 2014; Hagey et al., 2020; Ohtsuka and Kageyama, 2019; Salomoni and Calegari, 2010); however, the mechanism has remained elusive. The NSC resides in a glial niche and in close proximity to their lineages (Doe, 2008). Given that the cell-cycle blocked lineages have no or less progeny, we could not rule out the possibility that type II NSCs with cell cycle arrest failed to undergo normal temporal progression indirectly due to a lack of feedback signaling from their progeny. To address this concern, we generated random single NSC clones driving *Cd-k1*RNAi, which developed alongside other wild-type type II NSCs. If the NSCs are still delayed in their temporal transition despite being in the same environment as wild-type NSC lineages, we can conclude that the cell cycle plays an independent role in the temporal progression of these NSCs. We found that, indeed, all type II NSC clones failed to switch from early Imp expression to EcR and Syp expression in late developmental stages (Figure 2A–F), demonstrating the intrinsic ‘clock’ that NSCs have in regulating their TTF progression independent of animal development. However, we cannot rule out the lineage-specific roles of progeny in providing feedback signals to regulate the timing of the temporal gene expression switch. When the cell cycle was blocked 42h ALH, NSCs failed to transition from early to late temporal gene expression. This suggests that early-born progeny do not influence the temporal gene expression of NSCs. During cortical development, both cell cycle and extrinsic factors govern neuronal fate, and it was proposed that neuronal progenitors undergo cyclic changes in their ability to respond to extrinsic cues (McConnell and Kaznowski, 1991; Okamoto et al., 2016). Further studies are needed to explore whether lateborn progeny regulate temporal gene expression by providing feedback signals to the parent NSC.

## Materials and Methods

### Immunohistochemistry

Larval brains were dissected in PBS and mounted on poly-D-lysine-coated coverslips. Samples were fixed for 23 minutes in 4% PFA in PBST, and then washed in PBST for 3x20 minutes. Following, the samples were blocked with 2% normal donkey serum and 2% normal goat serum (Jackson ImmunoResearch Laboratories, Inc.) in PBST for 40 minutes at room temperature. Samples were then incubated in a primary antibody mix diluted in PBST overnight or for 1–2 days at 4°C. Primary antibodies were removed, and samples were thoroughly washed with PBST. Samples were then incubated in secondary antibodies for 2 hours at room temperature. Secondary antibodies were removed, and samples were washed in PBST. Samples were dehydrated with an ethanol series of 30%, 50%, 75%, and 100% ethanol, then incubated in xylene (Fisher Chemical X5–1) for 2x10 minutes. Samples were mounted onto slides with DPX (Sigma-Aldrich 06552) and cured for 3–4 days, then stored at 4°C until imaged.

### Image acquisition and analysis

Fluorescent images were acquired on both Zeiss LSM 710 and 800. Scale bars were given from all stacks within maximum intensity projection images. Brightness and contrast were adjusted in images uniformly for better visualization across the entire image across control and experimental samples. Statistics and bar graphs were computed using Prism 10.

### Figure Preparation

Confocal images were prepared using FIJI or Imaris 10.0.0. Figures and schematics were assembled using Adobe Illustrator (2025).

### Generation of clones

To generate RNAi clones, *hsFLP;10xUASmc-d8::GFP; ActFRTstopFRTGAL4* flies were crossed to the RNAi lines for the gene of interest or control *UAS-mCherryRNAi* line. Embryos were collected over a period of 4 hr. After hatching, larvae were collected and then heat shocked in a 37°C water bath for 8 minutes and reared at 25°C until the desired time point.

## Supplementary Material

Supplement 1

## Figures and Tables

**Resource Table:** 

Antibodies	Source	Identifier
Chicken anti-GFP (1:1500)	Aves Labs	Cat# AB_2314866; RRID_AB_2314866
Rabbit anti-Asense (1:1000)	Chien-Yu Lee	N/A
Rat anti-IMP (1:200)	Claude Desplan	N/A
Rabbit anti b -GAL (1:1000)	MP Biomedicals	Cat# 559762
Mouse anti-EcR-B1 (1:2000)	Carl Thummel	N/A
Guinea pig anti-Syncrip (1:2000)	Ilan Davis	N/A
**Chemicals**		
16% Paraformaldehyde	Electron Microscopy Sciences	Cat# 15710
Triton X-100	Sigma-Aldrich	Cat# T8787
Apple Juice	S. Martinelli & Co	N/A
Schneider’s Insect medium	Sigma-Aldrich	Cat# S0146
Agar	Sigma-Aldrich	Cat# A1296
DPX mounting medium	Sigma-Aldrich	Cat# 06522
Sucrose	Research products International	Cat# 57-50-1
Xylene	Fisher Scientific	Cat# 1330-20-7, 100-41-4
**Strains**		
hsFlp,UASGFP;ActFRTstop FRTGAL4	This study	NA
UAS-pavRNAi	BDSC	BDSC_43963
UAS-CdklRNAi	BDSC	BDSC_36117
UAS-mCherryRNAi	BDSC	BDSC_35785
WorGAL4,AseGal80; 20xUASmcd8:GFP	Chris Doe	NA
Svp-LacZ	BDSC	BDSC_26669
Software and Algorthms		
Imagej	Fiji	Version: 2.9.0/1.53t
Adobe Illustrator (version 24.005.20307)	Adobe Systems	https://www.adobe.com/products/illustrator.html
GraphPad Prism 9	GraphPad Software	https://www.graphpad.com/
Imaris v9.9 and above	Oxford Instruments	RRID: SCR_007370; https://www.oxinst.com/news/imaris-launches-version-9.9-with-machine-learning-and-open-source-connections

**Fly Genotype with Associated Figures:** 

Experimental line	Main	Supplementary
hsFlp,UASGFP;ActFRTstopFRTGAL4 crossed to UAS-mCherryRNAi	Fig. 2A, 2B, 2C	Fig. S1A
hsFlp,UASGFP;ActFRTstopFRTGAL4 crossed to UAS-Cdk1RNAi	Fig. 2D, 2E, 2F,	Fig. S1B
hsFlp,UASGFP;ActFRTstopFRTGAL4 crossed to UAS-pavRNAi	Fig. 2G, 2H, 2I	Fig. S1C
WorGal4,AseGal80; 20xUASmcd8:GFP crossed to Svp-LacZ	Fig. 3A	
WorGal4,AseGal80; 20xUASmcd8:GFP crossed to UAS-Cdk1-RNAi;Svp-LacZ	Fig. 3B	
hsFlp,UASGFP;ActFRTstopFRTGAL4 crossed to UAS-mCherryRNAi	Fig. 4A, 4B, 4C	
hsFlp,UASGFP;ActFRTstopFRTGAL4 crossed to UAS-Cdk1RNAi	Fig. 4D, 4E, 4F	
hsFlp,UASGFP;ActFRTstopFRTGAL4 crossed to UAS-pavRNAi	Fig. 4G, 4H, 4I	
hsFlp,UASGFP;ActFRTstopFRTGAL4 crossed to UAS-mCherryRNAi	Fig. 5A, 5B, 5C, 5K, 5L, 5M	
hsFlp,UASGFP;ActFRTstopFRTGAL4 crossed to UAS-CdklRNAi	Fig. 5D, 5E, 5F, 5N, 5O, 5P	
hsFlp,UASGFP;ActFRTstopFRTGAL4 crossed to UAS-pavRNAi	Fig. 5G, 5H, 5I, 5Q, 5R, 5S	
