# Supplementary material for: Cell cycle-dependent cues regulate temporal patterning of the Drosophila central brain neural stem cells
Source: bioRxiv. 2025 Jul 2:2025.01.16.629716. Originally published 2025 Jan 16. Preprint. [Version 2] doi: 10.1101/2025.01.16.629716 (PMC11760265; doi:10.1101/2025.01.16.629716)
Supplement: Supplementary file 1 [file NIHPP2025.01.16.629716V2-supplement-1.pdf]

bioRxiv preprint doi: https://doi.org/10.1101/2025.01.16.629716; this version posted July 2, 2025. The copyright holder for this preprint (which was not certified by peer review) is the author/funder, who has granted bioRxiv a license to display the preprint in perpetuity. It is made available under aCC-BY 4.0 International license.

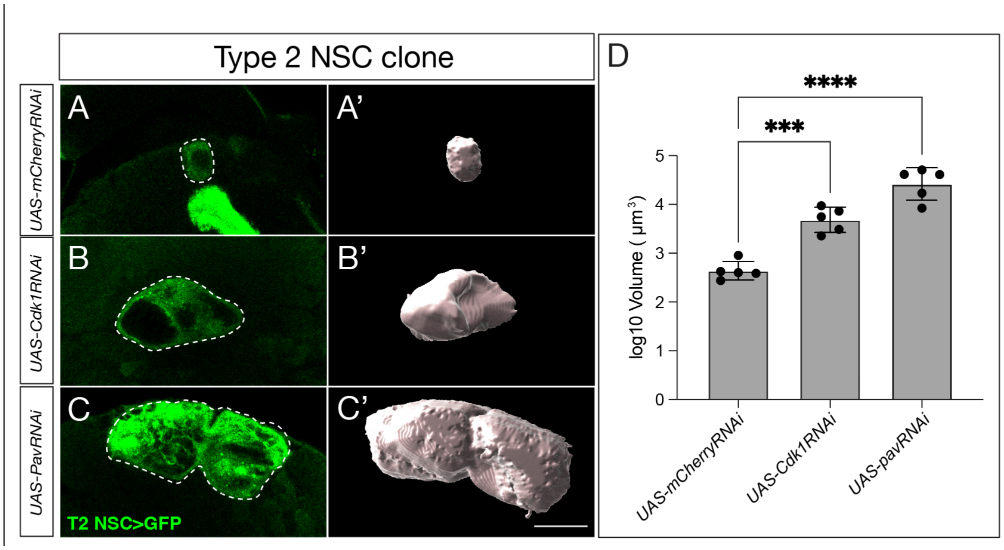

Figure S1: *pav* and *Cdk1*RNAi type 2 NSC clones are larger in volume. Confocal scans with their respective Imaris reconstruction are shown on the right for mCherryRNAi (A-A'), Cdk1RNAi (B-B'), and pavRNAi (C-C') clones. (D) Quantification of $\log^{10}$-transformed clone volumes (μm^3); n = 5 clones per condition, plotted as mean ± SEM. Overall treatment effect by Welch's ANOVA, $p < 0.0001$. Post-hoc Dunnett's T3 vs. mCherryRNAi: Cdk1RNAi, $p = 0.0003$; pavRNAi, $p < 0.0001$. only one clone was quantified per animal. Scale bar represents 20um.

bioRxiv preprint doi: https://doi.org/10.1101/2025.01.16.629716; this version posted July 2, 2025. The copyright holder for this preprint (which was not certified by peer review) is the author/funder, who has granted bioRxiv a license to display the preprint in perpetuity. It is made available under aCC-BY 4.0 International license.

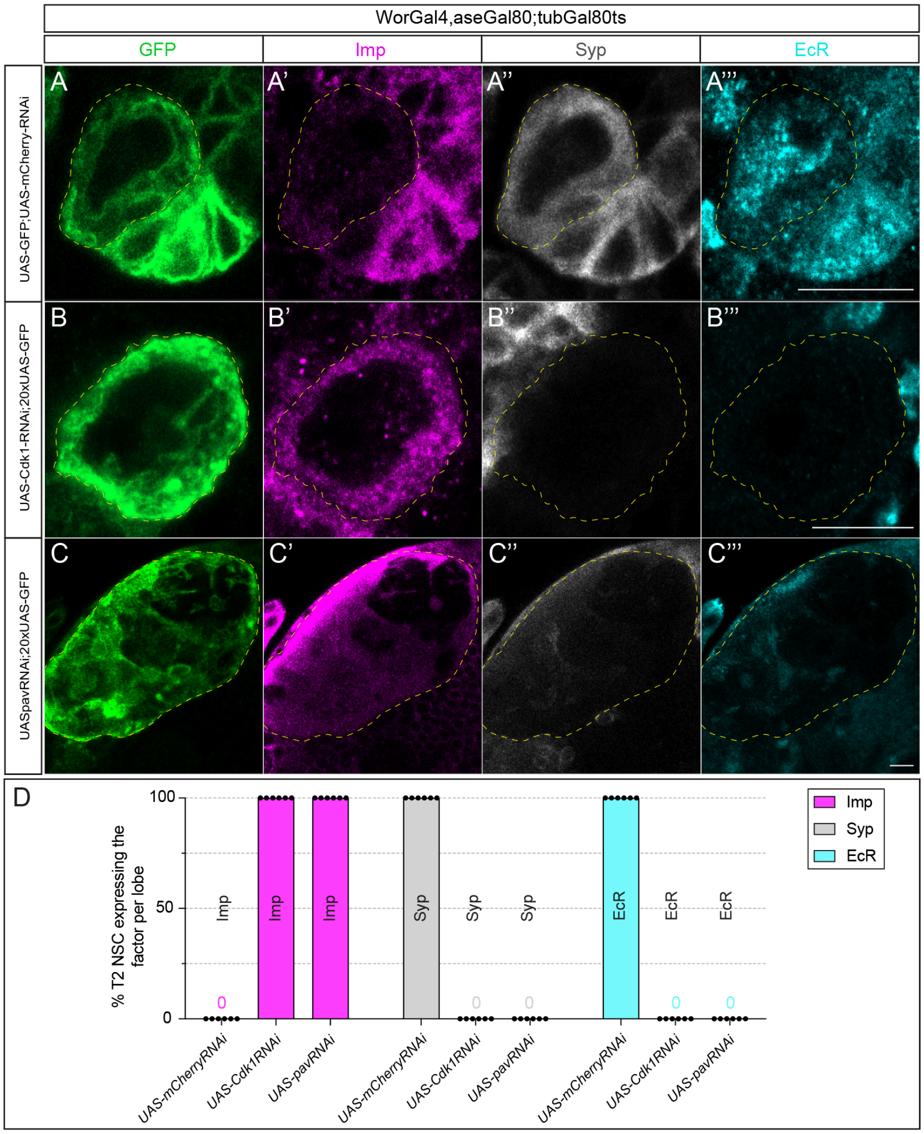

Figure S2: Cell-cycle and cytokinesis inhibit the early-to-late transition of temporal factors in type 2 NSCs. (A-A''') Type 2 NSC control UAS-mCherryRNAi (circled) at 72h ALH show normal temporal gene expression progression. Early factor Imp is off, and late factors Syp and EcR are on at 72h ALH (B-B''''). Cdk1RNAi type 2 NSCs fail to downregulate early factor Imp, and late factors Syp and EcR are not turned on. (C-C''') Similarly, cytokinesis-blocked type 2 NSCs using pavRNAi fail to downregulate Imp and upregulate Syp and EcR. (D) Quantifications of early and late temporal factor expression in control, cell cycle, and cytokinesis blocked type 2 NSCs; n=6 for each genotype. Type 2 NSCs are identified as large cells expressing UAS-mcd8::GFP. Scale bars represent 10um.
