# Supplementary material for: Cell cycle-dependent cues regulate temporal patterning of the Drosophila central brain neural stem cells
Source: bioRxiv. 2025 Jan 16:2025.01.16.629716. Preprint. [Version 1] doi: 10.1101/2025.01.16.629716 (PMC11760265; doi:10.1101/2025.01.16.629716)
Supplement: Supplementary file 1 [file NIHPP2025.01.16.629716v1-supplement-1.pdf]

bioRxiv preprint doi: https://doi.org/10.1101/2025.01.16.629716; this version posted January 16, 2025. The copyright holder for this preprint (which was not certified by peer review) is the author/funder, who has granted bioRxiv a license to display the preprint in perpetuity. It is made available under aCC-BY 4.0 International license.

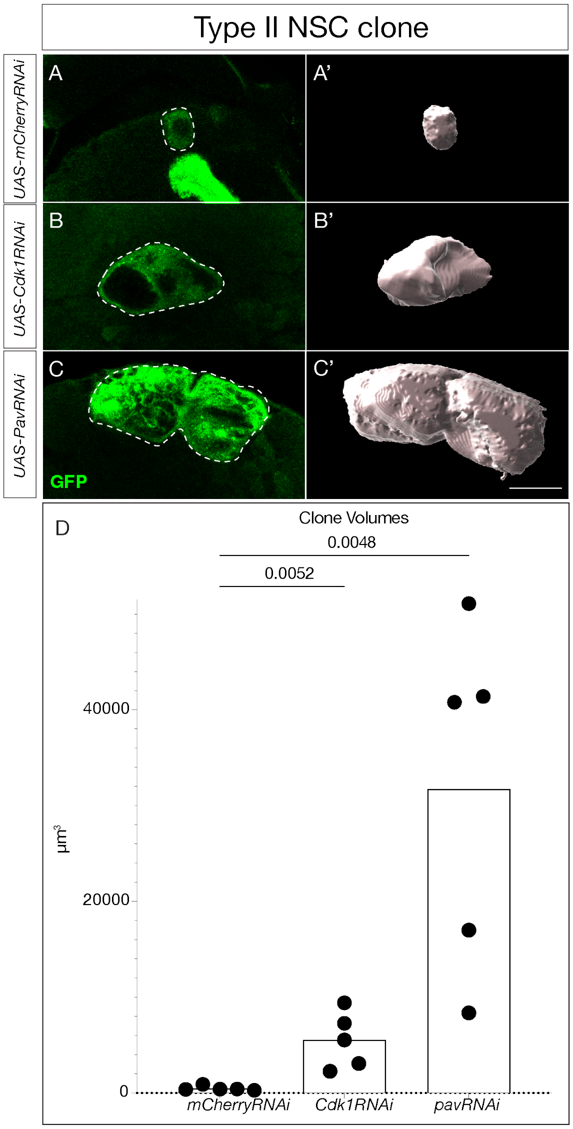


**Figure S1: *pav* and *Cdk1*RNAi type II NSC clones are larger in volume.** Confocal scans with their respective imaris reconstruction are shown on the right for mCherryRNAi (A-A'), Cdk1RNAi (B-B'), and pavRNAi (C-C') clones. (D) Quantification of clone volumes. Statistics: t-test p= 0.0052 for mCherry-Cdk1 and p= 0.0048 for mCherry-pavarotti, n=5 clones.
